# Fungal Peritonitis: Underestimated Disease in Critically Ill Patients with Liver Cirrhosis and Spontaneous Peritonitis

**DOI:** 10.1371/journal.pone.0158389

**Published:** 2016-07-08

**Authors:** Tobias Lahmer, Andreas Brandl, Sebastian Rasch, Roland M. Schmid, Wolfgang Huber

**Affiliations:** 1 II. Medizinische Klinik und Poliklinik, Klinikum rechts der Isar der Technischen Universität München, Munich, Germany; 2 Department of General, Visceral and Transplantation Surgery and Department of General, Visceral, Vascular and Thoracic Surgery, Campus Virchow and Mitte, Charité, Universitätsmedizin Berlin, Berlin, Germany; University of Leicester, UNITED KINGDOM

## Abstract

**Introduction:**

Spontaneous peritonitis, especially spontaneous fungal peritonitis (SFP), is an important and potentially fatal complication in patients with endstage liver disaese. We evaluated potential risk factors, microbiological findings, and outcome of patients with SFP compared to spontaneous bacterial peritonitis (SBP) in critically ill patients.

**Methods:**

Retrospective analyses of critically ill patients with suspected spontaneous peritonitis.

**Results:**

Out of 205 patients, 20 (10%) had SFP, 28 (14%) had SBP, 48 (24%) had peritonitis without microbiological findings (SP) and 109 (52%) had no-peritonitis (NP). APACHE II and SOFA score were significantly higher in patients with SFP (26; 22–28; p<0.004 and 16; 14–18; p<0.002), SBP (26; 22–28; p<0.004 and 16; 14–18; p<0.002) and SP (24; 18–30; p<0.045 and 14; 10–18; p<0.044) as compared to NP (22; 16–24 and 12; 10–14). CHILD Pugh classification was mainly CHILD C and MELD Score was in patients with SFP (34; 18–40; p<0.001), SBP (32;12–40 p<0.002) and SP (29; 14–40 p<0.003) significantly higher as compared to NP (25;8–40). Nosocomial peritonitis could be significantly more often found in patients with SFP (65%; p<0.023) and SBP (62%, p<0.030) as compared to SP (51 p = 0.243) and NP (45%). Antibiotic pretreatment last 3 month prior peritonitis was significantly more often in patients with SFP (85%; p<0.002), SBP (71%, p<0.033), and SP (56; p<0.040) as compared to NP (33%). *Candida albicans* (60%; 12/20) was the most common isolated fungus, followed by *Candida glabrata* (13%) and *Candida krusei* (13%). Mortality rate was significantly higher in patients with SFP (90%, p<0.001), followed by SBP (75%; p<0.001) and SP (69%; p<0.001) as compared to NP (45%).

**Conclusion:**

SFP is not a rare complication in end stage liver disease which is associated with increased mortality. Physicians should be aware of SFP in patients with CHILD C liver cirrhosis, elevated MELD score, antibiotic pretreatment and nosocomial peritonitis.

## Introduction

Spontaneous peritonitis is an important and potentially fatal complication in patients with endstage liver disease with mortality rates of up to 40% for every single episode [1]. Typically, spontaneous bacterial peritonitis (SBP) is most commonly caused by enteric bacteria from the gastrointestinal tract and potential risk factors are well studied [1,2].

However, the combination of liver cirrhosis and critical illness is been discribed as a relevant combination causing aquired immunodeficiency [3]. This is not only expressed in increasing morbidity and mortality rate but also in increasing findings of spontaneous fungal peritonitis (SFP) [4].

SFP is an understimated disease, especially in critically ill patients. Little is known about the clinical course, typical fungal isolates and outcome in critically ill patients with SFP [4].

To our knowledge this retrospective study is the largest analyses of critically ill patients evaluating potential risk factors, microbiological findings, and the outcome of patients with SFP compared to SBP in critically ill patients.

## Material and Methods

### Patients

This retrospective study was conducted at the medical ICU of the University Hospital Munich, Germany. We analyzed the medical records of all patients with liver cirrhosis and spontaneous bacterial peritonitis that were treated at the ICU between January 2010 and December 2014. This observational retrospective study without any specific intervention was reviewed and approved by the ethics committee of Klinikum rechts der Isar, TU München, of our university hospital, and all data were processed anonymously. Need for informed consent was waived for this retrospective analysis. In patients who were admitted more than once to the ICU during the study period, only the first episode of ICU stay was analyzed.

Data regarding the patients clinical and demographic characteristics were documented according to the medical record and the electronic patient database of our hospital.

### Definitions and Methods

Liver cirrhosis was diagnosed either by histological specimen, by ultrasound and/ or computed tomography and by clinical criteria for instance ascites or esophageal varices.

All patients underwent diagnostic paracentesis immediately after admission or upon presentation of signs and/or symptoms suggestive of peritonitis. Ten milliliters of ascitic fluid were collected at the bedside and inoculated into aerobic and anaerobic blood culture bottles.

Bacterial culture was performed manually using MacConkey and blood agar plate. Fungal culture was performed in the same manner using a Schaedler broth (Becton Dickinson) and Sabouraud dextrose agar slant (Becton Dickinson). The following diagnostic criteria were determined for spontaneous peritonitis: polymorphonuclear leukocyte (PMN) count >250 cells/mm3 in ascitic fluid, positive microbiological findings thru culture, exclusion of intra-abdominal surgical procedure or endoscopic biliary intervention during the past 4 weeks, exclusion of malignancy. If spontaneous peritonitis was diagnosed after 48h after hospital admission, it was classified as noscomial.

Empirical antibiotics were administered after diagnostic paracentesis if diagnostic criteria were fulfilled. Paracentesis was repeated after 48 h and treatment was considered as successful if PMN count was <50% of the initial value.

For definition of fungal disease we used the criteria proposed by the European Society of Clinical Microbiology and Infectious Diseases (ESCMID) guideline for the diagnosis and management of Candida diseases 2012 [5].

### Statistics

We used IBM SPSS Statistics 23 (SPSS inc., Chicago, IL, USA) for all statistical analyses in this study. To present descriptive statistics, we calculated mean ± standard deviation for normally distributed continuous data, and absolute and relative frequencies for categorical

Data (Fishers exact test). To compare the other variables, we performed the t-test for paired samples and the Wilcoxon signed rank test for paired samples for normally distributed data and not normally distributed data, respectively. Multivariate logistic regression analysis was performed by backward selection procedures to identify predictors of death using variables identified univariate analysis (p<0.10). A p-value below a significance level of 5% (p < 0.05) indicates statistical significance.

## Results

We analyzed the medical records of all patients with liver cirrhosis and spontaneous peritonitis that were treated at our ICU between January 2010 and December 2014.

Out of these 205 patients we identified 20 (10%) patients with spontaneous fungal peritonitis (SFP), 28 (14%) patients with spontaneous bacterial peritonitis (SBP), 48 (24%) patients with peritonitis without microbiological findings (SP) and 109 (52%) patients with no-peritonitis (NP). This means a total of 96 (47%) patients had spontaneous peritonitis. The demographic and clinical characteristics of the patients are presented in Table 1.

**Table 1:** Demographic and clinical characteristics of the patients.

	All Patients (n = 205)	Patients without Peritonitis (n = 109)	Patients with peritonitis without microbiological findings (n = 48)	p-value	Patients with spontaneous *bacterial* peritonitis (n = 28)	p-value	Patients with spontaneous *fungal* peritonitis (n = 20)	p-value
Gender (male/%)	58	56	60	p = 0.548	58	p = 0.458	58	p = 0.543
Age (y)	57;23–83; 11	57;23–83; 11	58;36–81; 12	p = 0.433	55;27–78;12	p = 0.344	58;46–78;8	p = 0.455
APACHE II Score	25;18–30;3	22; 16–24; 2	24; 18–30; 3	**p<0.045**	26; 22–28; 2	**p<0.004**	26; 22–28;3	**p<0.004**
SOFA Score	15;10–18;2	12; 10–14; 2	14; 10–18; 2	**p<0.044**	16; 14–18; 1	**p<0.002**	16; 14–18; 1	**p<0.002**
**Etiology of cirrhosis n (%):**								
Heavy alcohol intake	179; (87)	94;(87)	45;(94)		21;(75)		18;(90)	
HBV infection	1; (0,5)	0; (0)	0;(0)		1; (4)		0; (0)	
HCV infection	8; (4)	5; (4)	0;(0)		3; (11)		0; (0)	
Biliary cirrhosis	4; (2)	2; (2)	1;(2)		0; (0)		0; (0)	
Unknown	13; (6,5)	8; (7)	2;(4)		3; (11)		2;(10)	
Nosocomial peritonitis, (%)	67	45	51	P = 0.243	62	**p<0.030**	65	**p<0.023**
Antibiotic pre-treatment last 3 months n; (%)	101; 49%	37; (33)	27; (56)	**p<0.040**	20; (71)	**p<0.033**	17; (85)	**p<0.002**
MELD score	27; 8–40; 8	25;8–40;7	29; 14–40; 7	**p<0.003**	32;12–40;7	**p<0.002**	34; 18–40; 8	**p<0.001**
**Child-Pugh-class (n;%):**								
A	0; (0)	0; (0)	0;(0)		0;(0)		0;(0)	
B	29;(14)	25; (21)	3;(6)		1;(4)		0;(0)	
C	176;(86)	84; (79)	45;(94)		27;(96)		20;(100)	
Serum albumin (g/dl)	2,9; 1,4–4,6; 0,6	3; 1,4–4,6; 0,6	3; 1,6–4,2; 0,6	p = 0.655	3;1,7–4,1;0,6	p = 0.467	2,6;1,4–3,9;0,6	p = 0.144
Serum bilirubin (mg/dl)	9,9;0,2–43,1; 10,2	8,3; 0,2–42,6;10	11,1; 0,5–43,1;9,9	**p<0.023**	14;0,8–36,7;10,9	**p<0.005**	15,2; 1,6–35,4;8,8	**p<0.001**
Serum creatinine (mg/dl)	2; 0,4–6,6; 1,2	1,8; 0,4–4,7; 0,9	2,4; 0,7–6,6;1,4	p = 0.133	2,2; 0,5–5,9;1,4	p = 0.158	2,1; 0,7–5,4;1,5	p = 0.243
INR	1,9; 0,6–7; 0,9	1,8; 0,6–5,6; 0,8	2,2; 0,9–7; 1,2	p = 0.188	2,3;1,2–5,4;1,1	p = 0.202	2,1; 1,2–5,4;1	p = 0.344
Leukocyte count G/l	12,4; 0,9–39,7; 7,9	10,4; 0,9–33,6; 6,3	14,9; 1,9–39,7; 9,4	p = 0.060	14,1;0,9–31,5;8,7	p = 0.188	17,6;3,1–31,5;9,2	p = 0.055
C-reactive protein mg/dl	5,1; 0,1–37,4; 5,9	3,7; 0,1–32,5; 5	7,2; 0,4–37,4; 7,6	p = 0.055	6,9; 0,3–24;5,1	p = 0.256	6,4;0,4–24;5,8	p = 0.100
Procalcitonin ng/ml	3,3; 0,1–72,3; 8,6	1,6; 0,1–27,7; 3,7	4,1; 0,1–72,3; 10,8	p = 0.101	9,5;0,1–64,1	p = 0.088	3,8;0,5–17,7;4,4	p = 0.099
Percentage of peritonitis (%)			23		14		10	
ICU stay (d)	23+-6	19+-4	24+-5	**p<0.030**	24+-4	**p<0.030**	25+-6	**p<0.020**
Mortality rate (%)	58	45	69	**p<0.001**	75	**p<0.001**	90	**p<0.001**

Acute Physiology And Chronic Health Evaluation (APACHE), Sequential Organ Failure Assessment score (SOFA), model of endstage liver disease (MELD). Data are presented as mean ± standard deviation, statistical significant parameters are highlighted.

A high overall APACHE II and SOFA score could be observed in this study population which expresses the severity of the underlying disease.

APACHE II and SOFA score were significantly higher in patients with SFP (26; 22–28; p<0.004 and 16; 14–18; p<0.002), SBP (26; 22–28; p<0.004 and 16; 14–18; p<0.002) and SP (24; 18–30; p<0.045 and 14; 10–18; p<0.044) as compared to patients with NP (22; 16–24 and 12; 10–14).

Main cause of end stage liver disease was in all groups heavy alcohol intake, followed by hepatitis C and B, biliary cirrhosis and unknown reasons (see Table 1). This is in the line with the CHILD Pugh classification which was in all groups mainly CHILD C (see Table 1).

MELD (model of endstage liver disease) Score was in patients with SFP (34; 18–40; p<0.001), SBP (32; 12–40 p<0.002) and SP (29; 14–40 p<0.003) significantly higher as compared to patients with NP (25;8–40).

Serum bilirubin was significantly altered in patients with SFP (15,2; 1,6–35,4 p<0.001), SBP (14; 0,8–36,7; p<0.005), SP (11,1; 0,5–43,1 p<0.023) as compared to patients with NP (8,3; 0,2–42,6). The other laboratory parameters albumin, creatinine and INR were not statistically signficant altered in the different groups.

*Candida albicans* (60%; 12/20) was the most common fungus isoltated from ascites cultures, followed by *Candida glabrata* (13%), *Candida krusei* (13%); *Candida Kefyr (9%*); *Candida parapsilosis* (4%) and *Candida tropicalis* (4%) (see Table 2). *Fusarium spp*. could be isolated in 4%.

**Table 2:** Microbiological findings.

	Microbiological Findings	
Patients with spontaneous ***bacterial*** peritonitis n; (%)	***Gram positive bacterials*:**	
	*Staph*. *Epidermidis*	2; (6)
	*Staph*. *Aureus*	2; (6)
	*E*. *faecium*	6; (18)
	*Strept*. *mitis*	1; (3)
	***Gram negative bacterials*:**	
	*E*. *coli*	10; (33)
	*Serratia marcescens*	3; (9)
	*Kleb*. *Pneumoniae*	3; (9)
	*Veillonella spp*	1; (3)
	*Past*. *multocida*	1; (3)
	*Citrobacter freundii*	1; (3)
	*Pseudomonas stutzeri*	1; (3)
	*Hafnia alvei*	1; (3)
Patients with spontaneous ***fungal*** peritonitis n; (%)	*Candida albicans*	12; (52)
	*Candida glabrata*	3; (13)
	*Candida krusei*	3; (13)
	*Candida Kefyr*	2; (9)
	*Candida parapsilosis*	1; (4)
	Candida tropicalis	1; (4)
	*Fusarium spp*.	1; (4)

*E*. *faecium* (18%), *Staph*. *epidermidis* (6%) and *Staph*. *aureus* (6%) were the most common gram positive, *E*. *coli* (33%), *Serratia marcescens* (9%) and *Kleb*. *Pneumoniae* (9%) were the most common gram negative bacterials isolated from ascites fluid.

Nosocomial peritonitis could be significantly more often found in patients with SFP (65%; p<0.023) and in patients with SBP (62%, p<0.030) as compared to patients with SP (51 p = 0.243) and patients with NP (45%). Antibiotic pretreatment last 3 month prior peritonitis was significantly more often in patients with SFP (85%; p<0.002), SBP (71%, p<0.033), and SP (56; p<0.040) as compared to NP (33%). Antimycotic therapy was initiated in 30% oft he SFP patients. Leukocytes, C-reactive protein and Procalcitonin were not signficantly altered in the different groups.

An overall mean ICU stay of 23 days and a mortality rate of 58% could be observed.

This means in detail a significant different mean ICU stay of 25 days in patients with SFP (p<0.020), 24 days (p<0.030) in SBP, 24 days (p<0.030) in SP as compared to NP with 19 days.

Mortality rate was significantly higher in patients with SFP (90%, p<0.001), followed by SBP (75%; p<0.001) and SP (69%; p<0.001) as compared to NP (45%).

Risk factors for death included higher APACHE II, SOFA and MELD score. APACHE II score was independently associated with higher mortality rate using multivariate logistic regression (p = 0.031).

## Discussion

In our retrospective analyses we evaluated potential risk factors, microbiological findings, and the outcome of patients with SFP compared to SBP in critically ill patients.

Our results presented a higher mortality rate in patients with SFP as compared to patients with SBP, SP and NP. This is in the line with prior findings and underlines the severity of spontaneous fungal peritonitis in critically ill patients [6,7]. However, by several reasons such as delayed diagnosis or lack of clinical signs/awareness for SFP, only 30% of the patients included in this study were treated with antimycotics.

A total of 47% of the observed critically ill patients had spontaneous peritonitis. It is not surprising but disappointing that APACHE II is an independent risk factor for mortality. Thus, a more specific parameter focussing on risk factors for spontaneous peritonitis, especially fungal peritonitis, in critically ill patients with liver cirrhosis could not be observed. Thus, our data suggest that SFP is more often in patients with CHILD C liver cirrhosis and a MELD score beyond 30 points. Increased bilirubin levels, antibiotic pretreatment and nosocomial spontaneous peritonitis are other findings which are consistant with the risk for SFP. However, these findings could also be observed in patients with SBP and this might be a reason that awareness of fungal peritonitis is underrated.

Former studies described SFP as an infrequent and rare complication [8,9]. In contrast, in our collective 10% had SFP which is comparable to SBP with 14%. Moreover, these findings underline that fungal evidence in ascites fluid is not a rare complication and rather should be considered in end stage liver disease.

Most common species identified in SFP were *Candida albicans* and *Candida glabrata*, and gram negative bacterials such as *E*.*coli* in SBP. This is not surprising and in the line with pathphysiological findings in end stage liver disease which includes compromised intestinal barrier function due to oxidative stress and proinflammatory cytokines [10,11,12].

However, as reported in our study, 23% had defined spontaneous pertonitis without microbiological findings. Although, mortality rate is lower than in patients with SFP, these patients also die more often than patients without peritonitis.

It is well known that using routine culture methods sufficient microbiological findings could be detected in only 36–59%, and this may even be lower in detecting fungal species[13,14,15]. Thus, it is not known how many undected cases of fungal peritonitis are hidden in these findings.

As reported, standard laboratory parameters such as leukocytes, procalcitonin or C-reactive protein are to heterogenous for specific diagnosis of SFP. Therefore, the use of new diagnostic techniques such as PCR or the panfungal biomarker 1,3-Beta-D-Glucan may be a new approach in early indentification of SFP [16,17].

There are several limitations of this study: First, it´s retrospective nature which includes possible information bias. Second, our study was conducted at a single ICU. Finally, microbiological findings may not be representive and colonization or secondary peritonitis in SFP patients could not completley be excluded.

## Conclusion

SFP is not a rare complication in end stage liver disease. SFP is underrated and associated with increased mortality rate as compared to SBP. Physicians should be aware of SFP in patients with CHILD C liver cirrhosis, elevated MELD score, antibiotic pretreatment and nosocomial peritonitis.
